# Antibiotic-loaded bone cement spacer usage combined with membrane induction in infected gap non-unions: A case series

**DOI:** 10.12669/pjms.345.14569

**Published:** 2018

**Authors:** Xinwei Liu, Guocheng Ding, Dapeng Zhou, Liangbi Xiang

**Affiliations:** 1 Xinwei Liu, Department of Orthopedics, Rescue Center of Severe Wound and Trauma of PLA, General Hospital of Shenyang Military Area Command, Shenyang 110016, P. R. China; 2 Guocheng Ding, Graduate School, Dalian Medical University, Dalian 116044, P.R. China; 3 Dapeng Zhou, Department of Orthopedics, Rescue Center of Severe Wound and Trauma of PLA, General Hospital of Shenyang Military Area Command, Shenyang 110016, P. R. China; 4 Liangbi Xiang, Department of Orthopedics, Rescue Center of Severe Wound and Trauma of PLA, General Hospital of Shenyang Military Area Command, Shenyang 110016, P. R. China

**Keywords:** Infection, Membrane induction, Bone defect, Trauma, Fracture fixation

## Abstract

**Objective:**

To explore the clinical effects upon gap nonunion of antibiotic-loaded bone cement spacer combined with membrane induction on infected bone defects.

**Methods:**

The data of 16 patients with infected bone defects admitted in General Hospital of Shenyang Military Area Command from January 2009 to January 2011 were analyzed retrospectively. There were 12 males and 4 females aged between 24-63 years age (average 43.1 ± 9.7) who had received antibiotic laiden bone cement spacer treatment. Stage-1, debridement and anti-biotic treatment with intraoperative preparation of customized bone cement spacers (antibiotics and bone cement spacer) with or without internal or external fixation Stage-2, removal of spacer and repair of bone defects using membrane-induced technique and internal fixation at bone defects site.

**Results:**

Sixteen patients were followed up for 39-98 months, (67.2 ± 20.4) on average. All patients with infected bone defects were healed. X-ray showed that fractures had healed and the new bone formed at graft site was more radio opaque than that of adjacent bone segments. The healing time was 6 to 10 months, (7.4 ± 1.1) on average. There was no recurrence of infection or deformity.

**Conclusion:**

The antibiotic-loaded cement spacer can control the local infection while maintaining the limb length and increasing the stability, reducing the contracture of bone and soft tissue, creating conditions for subsequent repair and reducing the infection rate of bone defects.

## INTRODUCTION

Infectious bone defects are commonly disabling diseases and are mainly caused by high-energy trauma such as traffic injuries and firearm injuries. The open surgery for both open fractures of the limbs and fractures can lead to bacterial infections. Dead bone pieces can encourage mass reproduction of bacteria lead to bone sepsis, resulting in nonunion or delayed healing. The features include high infection rate, severely reducing adjacent joint function repeated treatment cycle and high morbidity. It has always been a major challenge to treat bone defects in the field of traumatic orthopedics.^1^-^3^ Acute trauma frequently devitalizes so much soft tissue and the injured bone fragments favoring bacterial growth. It remains an ever present problem for the orthopedics surgeon as to how to reduce the infection rate, disability, complications and shorten the treatment cycle. 4,5

In this study hospital records were analyzed retrospectively including into the study group the data of 16 patients with infectious bone defects admitted from January 2009 to January 2011. All patients had infected gap non unions of various bones leading to need for interim filling of the gap with customized antibiotic bone cement spacer. After an interval second stage of surgery was done to fill the gap with bone graft and definitive fixation of fractures was done. The purpose of reducing the infection rate and shortening the treatment cycle was studied so as to provide a new treatment mode for rapid treatment of such high-energy injuries of complex nature

## METHODS

### Baseline clinical data

This study was approved by the ethics committee of our hospital, and written consent has been obtained from all patients. There were 16 patients in this study, including 12 males and four females, aged from 24 to 63 years, (43.1 ± 9.7) on average. The noted causes of injury were traffic injury in eight cases, falls in five cases, falloff heavy objects in three cases. Less patients were found to have upper limb involvement in seven cases than lower limb in nine cases. The length of the defect was 2.5-6 cm, with an average of (4.4 ± 1.1) cm. All patients met the following inclusion criteria: repeated episodes of acute attack after open fractures; pus or small bone fragments from the sinus discharge, and sometimes visible sequestrum exposed through skin defect, rough bone surface detectable with sinus probe; irregular bone thickening; X-ray and CT confirmation of the continuity interrupted fracture consolidation of the affected area; confirmation of the existence of infection by bacteriological cultures. Exclusion criteria: age less than 18 years old; combined multiple fractures; complicated medical diseases (such as severe diabetes, vasculitis, etc.); smoking history patients; pregnancy or follow-up pregnancy; combined severe osteoporosis.

### Treatment methods

Stage-1 (debridement and antibiotic treatment along with antibiotic laden bone cement spacer insertion): Relevant laboratory examinations (especially bacterial culture + susceptibility test) were done; antibiotics as per culture results were given intravenously in appropriate doses. In the process of surgical exploration and debridement, necrotic tissues on the wound surface were completely removed, including sequestrum and cicatricial tissue with poor blood supply; the original internal fixation device was removed and replaced with external fixation or gypsum back slab; reduction was performed at the bone defect site as best as possible, with internal fixation and realignment of fracture The antibiotic-loaded bone cement spacer was planted at the gap and if the wound was large or had poor soft tissue coverage, vacuum sealing drainage was used to assist the attachment with negative pressure. Postoperative systemic and topical medication of sensitive antibiotics was given. At this stage, further debridement may be performed once or twice to control infection or conduct skin grafting to close the wound surface.

Preparation of antibiotic-loaded bone cement spacers: A five mL syringe barrel was taken at atmospheric pressure and room temperature in a sterile environment, its sharp spout was cut off, and reaming was conducted with a three mm screw tap; a three mm-diameter plastic tube was taken, with one end inserted into the syringe and fixed; the syringe was disinfected for standby use. The sensitive antibiotics, polymethylmethacrylate powder and monomer (formula: antibiotic 0.1 g, polymethylmethacrylate bone cement 2.0 g, monomer 1.0 mL) were weighed, and mixed by hand (polymethylmethacrylate monomer added into the antibiotics and bone cement powder mixture), and immediately filled into the syringe before the antibiotic-loaded bone cement solidified, and then the stylet was pushed to squeeze the antibiotic cement into the plastic tube, and the plastic tube was peeled after it solidified; then the long strip of antibiotic cement plug was taken out.

Stage-2 (membrane-induced repair of bone defects): After infection was under control, antibiotic-leaded bone cement spacer and chain beads were surgically removed for membrane induction. After six weeks, re-operation was performed on the membrane-induced area for bone fixation. Bone graft taken from Iliac crests and Acetabular file was used to obtain pulverized cancellous bone and cortical bone mixed granules. The induced bio-film was incised, and the antibiotic-loaded bone cement spacer was removed. Bone replacement by the bone graft granules was performed within the membrane, and internal fixation devices were replaced with new ones, such as steel, intramedullary nail prosthesis or even artificial arthroplasty to fix fractures. Conventional antibiotic were given postoperatively, and dressing was changed as required. Regular observation was made on the surgical area, including X-ray showing bone healing, inflammatory reaction, sinus formation and other complications.

### Evaluation criteria for therapeutic effects

Infectious bone defect healing: disappearance of systemic and local symptoms and signs, sinus healing, no sequestrum on X-rays, bone healing in continuity. It was noted if there was recurrence of discharge or other signs of infection were seen within next two years.

The patient had repeated redness, swelling and suppuration at the ulnar incision after operation, pain in forearm even

after 4 months of dressing changes, deformity and limited function, and the X-rays (A, B) were performed. At stage 1,

Debridement was performed at the ulnar fracture end, the antibiotic-loaded bone cement spacer (C) was planted, and the postoperative X-rays (D, E) were conducted. At stage 2, membrane-induced repair of bone defects was performed; the fracture was fixed with steel (F, G); the internal fixation (H, I) of titanium plate was locked; 8 months after operation, the X-ray showed that the infected bone defect was cured (J, K) and the affected limb was well (L, M).

## RESULTS

**Fig.1:**
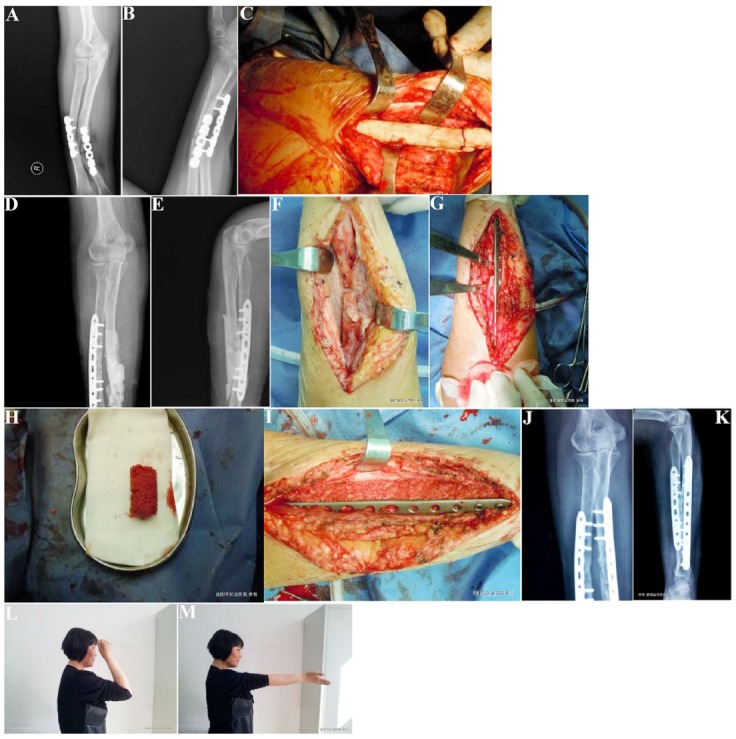
Treatment of a typical case. A female aged 43 years old suffered from open fracture in the right radius and ulna. The patient had repeated redness, swelling and suppuration at the ulnar incision after operation, pain in forearm even after 4 months of dressing changes, deformity and limited function, and the X-rays (A, B) were performed. At stage 1, Debridement was performed at the ulnar fracture end, the antibiotic-loaded bone cement spacer (C) was planted, and the postoperative X-rays (D, E) were conducted. At stage 2, membrane-induced repair of bone defects was performed; the fracture was fixed with steel (F, G); the internal fixation (H, I) of titanium plate was locked; 8 months after operation, the X-ray showed that the infected bone defect was cured (J, K) and the affected limb was well (L, M).

Sixteen patients were followed up for 39-98 months, (67.2 ± 20.4) on average. All patients with infected bone defects had been cured. One patient had recurrence of infection after membrane induction, with complications of pain, fever and serological examination suggest C reactive protein increased. After repeated debridement, the membrane was finally retreated. Bone mineral density at graft site changes in radio density on X-rays was significantly higher than that of preoperative one, with particulate bone resorption and new bone formation, proving that the bone fractures were healed. The healing time was 6 to 10 months, (7.4 ± 1.1) on average. There was no recurrence of infection or deformity (Supporting Information, Table-I). A typical case is shown in Fig.1.

**Table-I:** Demographics of the patients.

Patient number	Sex/ Age (y)	Localization	Fracture type	Microbiology	Defect size (cm)	Anti-infection spacer	Fixation	Bone union (month)	Follow-up (month)	Return to work
1	M/41	Humerus	Gustilo IIIB	MRSA	3.5cm	Vancomycin	Plate and screw	7.5	45	Yes
2	F/38	Tibia	Gustilo IIIA	MRSA	2.5cm	Vancomycin	Nail	7	62	Yes
3	M/49	Femur	Gustilo IIIA	Staph	6cm	Vancomycin	Plate and screw	8.5	71	Yes
4	M/51	Ulna	Gustilo II	Streptococcus	3.5cm	Vancomycin	Plate and screw	7	43	Yes
5	M/63	Tibia	Gustilo IIIB	Enterobacter	5cm	Gentamycin	Plate and screw	10	94	Yes
6	M/40	Tibia	Gustilo II	Staph+ Enterobacter	3.8cm	Gentamycin + vancomycin	Plate and screw	8.5	85	Yes
7	M/34	Tibia	Gustilo II	Streptococcus	3cm	Vancomycin	Nail	6.5	39	Yes
8	M/ 24	Tibia+Fibula	Gustilo IIIC	MRSA	4.5cm	Vancomycin	Plate and screw	7	92	Yes
9	F/45	Tibia	Gustilo IIIB	Staph	3.8cm	Vancomycin	Plate and screw	7	57	Yes
10	M/43	Tibia	Gustilo II	Staph	2.8cm	Vancomycin	Nail	6	61	Yes
11	M/52	Femur	Gustilo II	Staph	4.5cm	Vancomycin	Plate and screw	9	76	Yes
12	F/43	Radius+Ulna	Gustilo IIIB	Staph	4cm	Vancomycin	Plate and screw	8	42	Yes
13	M/33	Ulna	Gustilo IIIA	Enterobacter	5.5cm	Gentamycin	Plate and screw	6	98	Yes
14	M/37	Radius	Gustilo IIIB	Staph	3.5cm	Vancomycin	Nail	6.5	46	Yes
15	F/39	Ulna	Gustilo IIIB	Staph	5.8cm	Vancomycin	Plate and screw	6.5	81	Yes
16	M/57	Ulna	Gustilo IIIA	Enterobacter	6cm	Gentamycin	Plate and screw	8	83	Yes

MRSA: Methicillin-resistant S. aureus; M: male; F: female.

## DISCUSSION

For any successful treatment of infected bone defects, the core problem to be solved is to eliminate the infection and to let the bone heal without defect as soon as possible while ensuring reliable stability. Petersen et al.^6^ analyzed the results of bacterial culture for wound infection and found that the bacterial spectrum of infection involved a variety of bacteria such as gram-negative bacteria, staphylococcus epidermidis, aerobic bacteria and multiple resistant bacteria. Researchers have long emphasized the early (two hour after injury) intravenous usage of broad-spectrum antibiotics on open wounds to reduce the infection rate. Early use of antibiotics is particularly important in preventing infection, and sometimes bacterial culture is also necessary.^7^-^9^ For the treatment of infection combined with bone defect, we still follow the staged treatment of traditional chronic osteomyelitis.^10^ For a small range of bone defects (<4 cm), the clinical use of bone graft surgery for repair of bone defects may have a relatively good prognosis. For large bone defects (>4 cm), the most commonly used techniques at home and abroad include: 1) vascularized autologous bone grafts,^11^,^12^ such as the use of vascularized free fibula grafts to repair large segments of bone defects from various causes, but the shortcomings include a high demand for the level of microsurgical techniques, inaccurate effects of vascular anastomosis, long time of creeping substitution for bone graft, and repairable bone defects limited by the length and diameter of the donor area, as well as multiple complications in the recipient area, making it not an ideal procedure for infected bone defects. 2) The technique of distraction osteogenesis^13^-^15^ i.e. the Ilizarov Method firstly applied by Ilizarov in the Russia in 1950s, has now become a commonly used treatment for the repair of large segmental defects. Its disadvantages are a complicated operation, the requirement of special training/devices, multiple wire-related complications, long external fixation and poor patient tolerance. 3) The membrane induction technique,^16^-^18^ also known as the Masquelet technique has gradually gained popularity in Europe in recent years. It was initially used for chronic osteomyelitis, repair of large bone defects after tumor resection, with encouraging results.^19^,^20^ Induced membrane is a highly vascularized biofilm, which is rich in vascular endothelial growth factor, bone morphogenetic protein and many other growth factors. A good characteristic of Masquelet membrane is similar to the synovial tissue in morphology, with a thickness of around 0.5-2.0cm. The surface of the normal membrane is smooth, moist and continuous, while the common cause of failure is Infection has not been effectively controlled.^21^,^22^

Due to the aggressive operative management we interposed antibiotic laden bone cement spacers into the bone defect area during initial debridement in patients with infected bone defects. We presume that local infection was controlled through local release of sustained, effective and high concentrations of antibiotics, and at the same time, the spacers could maintain limb length and increase stability of the fracture end, reducing postoperative bone and soft tissue contractures The technique helped to create conditions for second stage definitive reconstruction.

In this study, we used the antibiotic-loaded bone cement spacer and the membrane-induced technique to the treatment of infected bone defects in limbs and obtained satisfactory results. However, more work still needs to be done, Future studies should compare it with the traditional techniques like tradional grafting or Ilizarov method. Masquelet membrane induction technique still needs further popularization in China. Based on large scale bacteriological studies the common infective agents and their strains should be identified. For patients with open fractures and bone defects, this technique will play a better role in the early treatment of infected bone defects. There is need to develop membrane inducement technology, such as the best timing of its application, as well as the composition of induction membrane and the mechanism of membrane induction, so as to provide a theoretical basis for the rapid treatment of infected bone defects.

### Author’s Contributions

**XL, GD & DZ**: Designed this study and prepared this manuscript.

**XL, GD & LX:** Performed this study.

**XL, GD & LX:** Collected and analyzed clinical data.
